# The Benefits of Low-Carbohydrate, High-Fat (LCHF) Diet on Body Composition, Leg Volume, and Pain in Women with Lipedema

**DOI:** 10.1155/2023/5826630

**Published:** 2023-11-18

**Authors:** Małgorzata Jeziorek, Angelika Chachaj, Monika Sowicz, Agnieszka Adaszyńska, Aleksander Truszyński, Justyna Putek, Krzysztof Kujawa, Andrzej Szuba

**Affiliations:** ^1^Department of Dietetics and Bromatology, Faculty of Pharmacy, Wroclaw Medical University, Wroclaw, Poland; ^2^Department of Angiology and Internal Medicine, Faculty of Medicine, Wroclaw Medical University, Wroclaw, Poland; ^3^Statistical Analysis Center, Wroclaw Medical University, Wroclaw, Poland

## Abstract

This study aimed to assess the potential benefits of a low-carbohydrate, high-fat (LCHF) diet on body composition, leg volume, and pain reduction in women with lipedema compared to overweight or women with obesity. The study included 113 female participants, 56 with lipedema and 57 with overweight/obesity (BMI >25 kg/m^2^) without lipedema. All subjects were prescribed a low-carbohydrate, high-fat (LCHF) diet with anti-inflammatory properties to adhere to for a duration of 7 months. Measurements of anthropometry, body weight, composition, and pain (VAS) were conducted at the study's commencement and conclusion. 52 participants completed the study. Both groups experienced a similar weight reduction, amounting to 12.9% compared to the baseline (−10.8 kg vs. −11.9 kg; *p* = 0.14, for lipedema and women with overweight/obesity, respectively). The most reduction was in body fat mass. Improvements in various parameters were observed, except for ankle circumferences, which decreased more in the lipedema group. Lipedema participants showed significantly reduced pain levels following the LCHF diet (4.6 ± 2.6 vs 3.0 ± 2.3; *p*  <  0.001). The LCHF diet holds promise for weight loss, body fat reduction, leg volume management, and pain alleviation in women with lipedema. These findings provide valuable insights into potential therapeutic strategies for lipedema management.

## 1. Introduction

Lipedema is a chronic and progressive condition that predominantly affects women. Clinical manifestations of lipedema include abnormal and disproportionate fat distribution between the legs and trunk, with adipose tissue accumulating symmetrically in the lower and/or upper extremities, sparing the hands and feet [1, 2].

The worldwide prevalence of lipedema was estimated to be approximately 11% [3, 4]. The disease often manifests during hormonal changes, such as puberty, pregnancy, or menopause [5]. The cause of lipedema remains unknown, but several hypotheses about its pathophysiology have been proposed: genetic predisposition, hormonal influence, fat cell changes, microvascular dysfunction, capillary damage, lymphatic disturbances, and inflammation [1, 5, 6]. Lipedema is often observed in familial clusters, suggesting a genetic component. It is thought to follow an autosomal dominant inheritance pattern with incomplete penetrance, with up to 60% of patients having affected first-degree relatives. Lipedema tends to manifest during hormonal changes and is believed to be influenced by estrogen. Changes in the distribution of estrogen receptors in fatty tissue may play a role. It is unclear whether lipedema involves an increase in the number of fat cells (hyperplasia) or enlargement of existing fat cells (hypertrophy). Studies suggest alterations in the initial stages of fat cell differentiation. Primary dysfunction in lymphatic and blood capillaries may result from hypoxia due to excessive fat tissue expansion, leading to endothelial dysfunction and increased angiogenesis. It may also be linked to mechanical issues in lymph drainage. Capillary damage may lead to a greater tendency to form hematomas and petechiae. Increased capillary permeability can cause tissue edema, initially compensated for by increased lymph drainage, but insufficiency may develop as the disorder progresses. Lymphatic scintigraphy shows early disturbances in lymphatic transport capacity. Capillary abnormalities can be worsened by issues in large blood vessels, like aorta stiffness, which may lead to vascular remodeling and hypertension. Dysregulation of the veno-arterial reflex (VAR) also contributes to edema and hematoma formation. Increased pain perception in lipedema may result from dysregulation of sensory nerve fibers due to inflammation. Mechanical compression of nerve fibers by fatty tissue does not explain the pain as it does not occur in other types of lipohypertrophy or lymphedema [5, 6].

Diagnosing lipedema may involve observing symptoms such as easy bruising and spontaneous or palpation-induced pain, which patients describe as burning, pressing, heavy, and increasing throughout the day. Affected individuals often experience sensations of heaviness and tightness in the affected extremities [7, 8]. Inflammatory changes in the adipose tissue may contribute to the pain, but the exact underlying mechanisms are still not fully understood [1].

Lipedema can be classified into four stages based on the severity of fat accumulation and skin changes. In the first stage (mild), fat accumulation is mild, and the skin appears smooth and symmetrical, with a slight increase in the size of affected areas. The second stage (moderate) exhibits more noticeable fat accumulation, resulting in a pear-shaped or column-like appearance of the legs, and the skin may develop small nodules. The third stage (severe) is characterized by a substantial increase in volume, larger nodules, and increased skin irregularities. In the fourth stage (very severe), lipedema can lead to significant deformities and functional limitations, with marked enlargement and fibrosis of the legs [5, 9, 10]. Additionally, lipedema is further classified into five types based on the distribution of fat and its appearance (type 1—buttocks, type 2—buttocks, hips, and thighs, type 3—buttocks, hips, thighs, and calves, type 4—arms, and type 5—calves) [5].

Individuals with lipedema often experience weight-related stigmatization and dissatisfaction with their bodies, leading to feelings of helplessness, self-stigmatization, depression, anxiety, stress, shame, and guilt [11, 12].

Conservative therapy for lipedema emphasizes promoting a healthy lifestyle, personalized weight control strategies, and graded activity training programs. As a significant proportion of lipedema patients are with overweight or obesity, dietary interventions play an essential role in management [1, 8, 13, 14]. Our previous research demonstrated that a low-carbohydrate, high-fat diet (LCHF) was more effective in lipedema treatment than diets with a low glycemic index [15]. However, the efficacy of any diet in lipedema treatment may be enhanced by its anti-inflammatory potential [16].

Studies have also suggested that LCHF diets can be beneficial for individuals with overweight or obesity, leading to decreased adiposity and appetite control through hormonal changes, including reduced levels of the hunger hormone leptin [17–20]. Some authors have reported a reduction in perceived pain in lipedema patients following an LCHF diet [21–23]. While the low-carbohydrate, high-fat (LCHF) diet has shown potential benefits for individuals with lipedema and patients with overweight/obesity, it is essential to be aware of potential nutritional deficiencies that may arise from this dietary approach. LCHF diets emphasize high-fat and low-carbohydrate food sources, which can lead to a reduced intake of certain nutrients, such as fiber, vitamins, and minerals [15, 24]. This study is notable for being the first to directly compare the effects of a low-carbohydrate, high-fat (LCHF) diet in individuals with lipedema to those dealing with overweight or obesity. Additionally, we thoroughly examined changes in both body composition and lower limb volume, a crucial aspect in evaluating the effectiveness of the diet for women with lipedema. The assessment of pain further confirms the benefits of the LCHF diet across various aspects.

Our study aimed to determine the potential benefits of a low-carbohydrate, high-fat (LCHF) diet on body composition, leg volume, and pain reduction in women with lipedema compared to women with overweight/obesity.

## 2. Materials and Methods

### 2.1. Study Design

This study encompassed a dietary intervention case-control design, characterized by its prospective nature spanning a duration of seven months. Notably, the study was conducted at a singular research center, ensuring controlled conditions for data collection and analysis.

### 2.2. Study Groups

**Figure 1:**
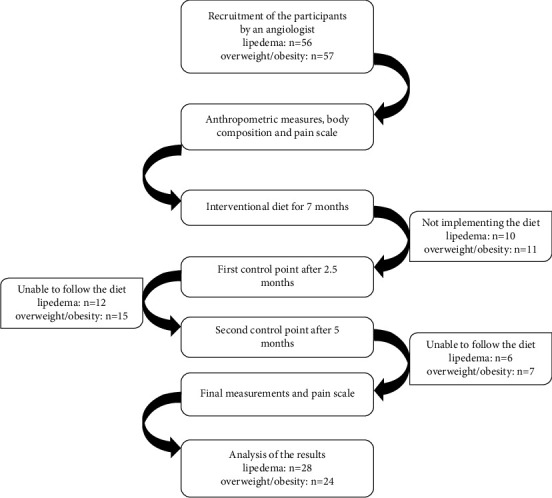
The scheme of the research.

The study enrolled a total of 113 female patients from the Angiology Outpatient Clinic at Wroclaw Medical University in Poland, including 56 with a diagnosis of lipedema based on typical clinical symptoms [10] and 57 with overweight or obesity (BMI >25 kg/m^2^) but without lipedema. Exclusion criteria included pregnancy, breastfeeding, a period of 6 months after pregnancy, various medical conditions such as lymphedema, edema in the course of chronic vein insufficiency or heart failure, diabetes mellitus, kidney or liver failure, hormonally unbalanced thyroid disease, cancer, and implanted devices.  Figure 1 demonstrates the scheme of the research. The study was conducted in accordance with the Declaration of Helsinki and approved by the Bioethics Committee at Wroclaw Medical University, Poland (KB-456/2019). Written informed consent was obtained from all participants. The study was initiated in January 2021 and concluded in May 2022. Of the original study population, 52 patients (28 in the lipedema group and 24 with overweight/obesity) completed the study. Lipedema patients were classified into 4 clinical stages and 5 types of the disease based on established criteria [5].

### 2.3. Measurement of Anthropometric and Body Composition Parameters

A TANITA HR-001 growth meter (Tanita, Japan) was used for height measurement. A TANITA MC-780MA (Tanita, Japan) was used for weight and body composition parameters. Parameters such as body fat percentage (%), body fat (kg), lean body mass (kg), total body water (kg), and visceral fat level were obtained. Patients were instructed not to consume food or drink for minimum 4 hours, not to engage in vigorous physical activity for 12 hours, and not to use diuretics for 6 hours prior to the study. Body mass index (BMI) was calculated as the ratio of body weight (kg) to height (m) squared. Waist, hip, thigh, calf, and ankle circumferences were measured with a standard tape measure to the nearest 1 cm. The mean value of the waist-hip ratio (WHR) was calculated as the ratio of waist to hip circumference. Leg circumferences were measured with a standard tape measure to the nearest 0.5 cm. The measurements were taken at 4 cm intervals from the ankle to the groin on the side of the leg. Based on these measurements, we calculated the volume of the leg (in milliliters) using the equation for a truncated cone [25].

### 2.4. Assessment of Pain Level in the Leg

The study employed validated method to assess pain levels—the visual analog scale (VAS). The visual analog scale is one of the most frequently used instruments to measure pain intensity. VAS is graphically represented by a graduated scale ranging from 0 (no pain at all) to 10 (full of pain), on which a patient indicates her perceived level of pain [26]. The VAS was assessed at baseline and after completing dietary intervention.

### 2.5. Dietary Intervention

The LCHF diet utilized a structure similar to a typical ketogenic diet, with less than 50 g of carbohydrates per day. The diet was designed with a Mediterranean style and focused on the quality of the food consumed, with many food products chosen for their anti-inflammatory properties. The daily energy intake was divided into 3 meals, consisting of a source of protein, fat, and vegetable additives. The sources of protein included eggs, cheese (such as Gouda, cheddar, feta, mozzarella, and full-fat quark), lean poultry, or beef, with the exclusion of animal protein sources such as pork, offal, and fatty skin poultry. The sources of fat included products high in MUFA (monounsaturated fatty acids), such as olive oil, avocado, olives, almonds, and hazelnuts, as well as PUFA (polyunsaturated fatty acids), such as canola and linen oil, walnuts, linen seeds, and oily marine fish such as salmon, herring, mackerel, and sardines. Each meal also included non-starchy vegetables such as tomato, cucumber, pepper, spinach, sprouts, cauliflower, zucchini, cabbage, radish, and aubergine, as well as one small portion (25−30 g) of berry fruits per day. The LCHF diet was also enriched with herbs and spices with anti-inflammatory properties, such as turmeric, cloves, garlic, thyme, rosemary, and black/green tea once a day. Additionally, in some cases, a daily snack consisting of nuts, seeds, or fresh vegetables was incorporated. The most detailed description of our LCHF diet we provided in our previous study [15].  Table 1 presents an exemplary 7-day dietary plan implemented among the subjects.

The diet plans were personalized for each participant by a clinical dietician using DietetykPro software (DietetykPro, Wroclaw, Poland) in order to increase adherence to the diet. The daily energy requirements for each participant were calculated based on their resting metabolic rate (RMR) and physical activity level (PAL) ratio. RMR was measured using the indirect calorimetry method with the Fitmate device (Cosmed, Rome, Italy) according to standard procedures [14]. PAL was determined individually for each patient, typically ranging from 1.3 to 1.5 [27]. The energy intake for each participant was set at 75–85% of their total energy requirements based on their body weight. The energy and nutritional status of diets was calculated based on the Polish Tables of Nutritional Value of Products and Dishes [28] and using the USDA (U.S. Department of Agriculture) food products database [29].

A 24 hour nutritional interview was conducted as part of regular monthly assessments, either through phone calls or personal visits. Specific intervals during the study, at 2.5 and 5 months, were also utilized for data collection. These measures provided valuable information regarding participants' adherence to the prescribed diet. This approach allowed us to identify common dietary mistakes, such as the consumption of carbohydrate-rich products, meal skipping, or unnecessary fat restrictions. Participants who did not comply with the prescribed diet were excluded to maintain the integrity of the study and ensure consistency of the intervention across the study group.

### 2.6. Statistical Analysis

Results are presented as mean values ± standard deviation or median and quartiles Q1 and Q3, when the data distribution was normal or non-normal, respectively. The conformity of the distribution in the given variable to the normal distribution was verified using the Shapiro–Wilk test. If the distribution significantly deviated from normal, we employed nonparametric tests, such as the Mann–Whitney *U* test for independent samples and the Wilcoxon test for repeated measurements. In cases where the distribution did not show a significant difference from normal, the *T*-test was used. To compare the differences between baseline and final anthropometric and body composition measurements, three types of tests were used: *T*-test, Wilcoxon, and Mann–Whitney *U* tests (depending on the results of the test checking normality of distribution and the test checking the homogeneity of variation). The Wilcoxon test was used to determine if there are differences in the VAS before and after dietary intervention. Results for all analyses were considered statistically significant when *p*  <  0.05. The power of the used tests was analyzed *posteriori*. Given an alpha level of 0.05 and a II-type error (beta) of 0.2, with final group sizes of 24 and 28, the effect sizes are as follows: (i) for the *t*-test for independent groups, *d* = 0.79; (ii) for the Mann–Whitney test, *d* = 0.81; (iii) for the Wilcoxon test for matched pairs, *d_z_* = 0.79. Here, *d* represents |*μ*_1_ – *μ*_2_|/*σ* for the *t*-test, *d_z_* represents |*μ_z_*|/*σ_z_* for the Wilcoxon test, where *μ*_1_ and *μ*_2_ are means in populations 1 and 2, *σ* is the standard deviation common to populations 1 and 2, *μ_z_* is the means of the differences between matched pairs, *σ_z_* is the standard deviation of the differences between matched pairs, and *d* is Cohen's effect size. STATISTICA v 13.0 from StatSoft Inc. (StatSoft Inc., USA) was used for statistical analysis of the results.

## 3. Results

The study found that the median age of participants in the patients with lipedema was 39.0 (33.0, 62.0) years, while in the women with overweight/obesity, it was 49.0 (41.5, 59.0) years; however, the difference between the two groups was not statistically significant (*p* = 0.14). However, at baseline, there were significant differences between the groups regarding waist and ankle circumferences, as indicated in Table 2. Additionally, the mean waist-to-hip ratio (WHR) was found to differ between the two groups, which is understandable given the disproportionate distribution of fat between the upper and lower body in lipedema females.

**Figure 2:**
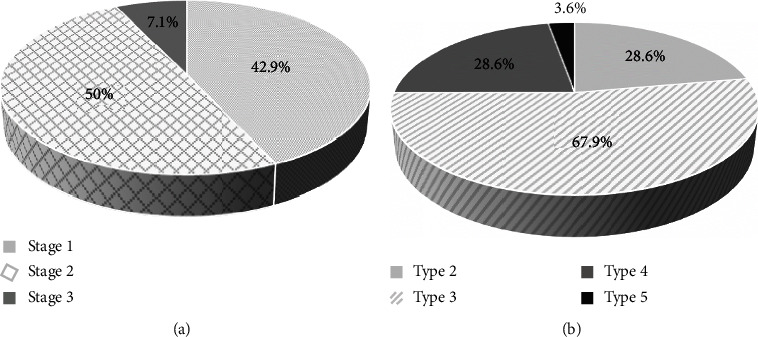
Prevalence of stages and types of lipedema in the study group (*n* = 28). (a) Stages of lipedema. (b) Types of lipedema. Some patients with lipedema in the study group had both type 2 or 3 (including the leg) and type 4 (including the arm) simultaneously. Therefore, the data in (b) exceed 100%. Detailed data regarding the percentage of patients with specific types of lipedema are as follows: type 2 (*n* = 5; 17.9%), type 2 and 4 (*n* = 3; 10.7%), type 3 (*n* = 14; 50.0%), type 3 and 4 (*n* = 5; 17.9%), and type 5 (*n* = 1; 3.6%).

The majority of women in the lipedema group were at stage 2, accounting for 50% of the group. Furthermore, type 3 lipedema was the most prevalent type, accounting for 67.9% of the group. The prevalence of lipedema stages and types in the study group is presented in Figure 2.

**Figure 3:**
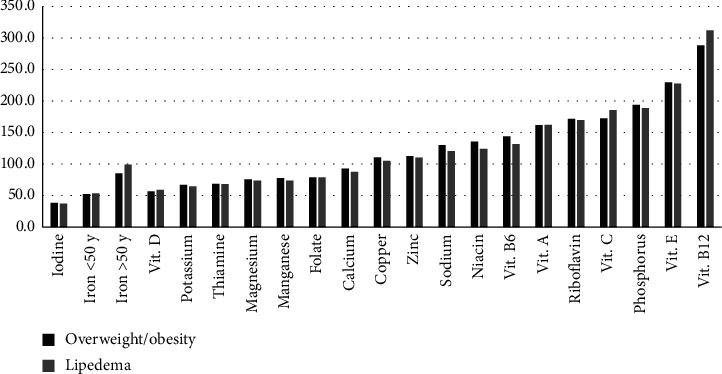
Compliance of vitamin and mineral intake with Polish recommendations (%) in study groups.

Despite individualized diet plans in both groups, the composition of the diets did not differ significantly. In both groups were deficit of a fiber, with the median intake being 34.4% and 35.2% of the daily recommendations for Polish adults in the lipedema and patients with overweight/obesity, respectively. Moreover, the diets in both groups were deficient in various essential nutrients, such as iron, magnesium, potassium, iodine, manganese, thiamine, calcium, folate, and vitamin D. The detailed diet composition in both groups is presented in Table 3. Compliance with the recommended intake of vitamins and minerals in the study groups is presented in Figure 3. The detailed comparison of vitamin and mineral intake with Polish recommended levels has been added to Table S1 in the supplementary materials.

After 7 months of following a low-carbohydrate, high-fat (LCHF) diet, participants in both groups experienced a reduction in body weight, with the majority of this reduction attributed to a decrease in body fat mass. Additionally, improvements in various anthropometric and body composition parameters were observed, with most of these improvements being statistically significant. Interestingly, there were no significant differences between the lipedema and patients with overweight/obesity, except for ankle circumferences, which decreased more significantly in the lipedema group. Additionally, total body water did not change significantly in the lipedema group, in contrast to the patients with overweight/obesity, where it decreased. A comparison of anthropometric and body composition measurements before and after dietary intervention in the study groups is presented in Table 4.

**Figure 4:**
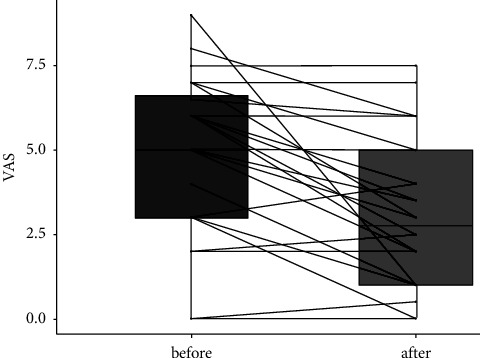
Change in pain level of each participant before and after dietary intervention in the lipedema group.

The study findings revealed a notable decrease in pain levels among participants with lipedema following a 7-month adherence to the low-carbohydrate, high-fat (LCHF) diet, as assessed by visual analog scale (VAS) scores (Table 4). The individual variations in perceived pain after implementing the LCHF diet are illustrated in Figure 4, demonstrating the impact of the dietary intervention on pain reduction in each participant.

## 4. Discussion

The objective of this study was to examine the potential benefits of a low-carbohydrate, high-fat (LCHF) diet on body composition, leg volume, and pain reduction in women with lipedema compared to women with overweight/obesity. The results of the study provide preliminary evidence supporting the viability of an LCHF diet as a treatment option for reducing leg volume and managing pain in individuals with lipedema.

The study's findings revealed significant improvements in various parameters following the implementation of the LCHF diet. Notably, the women with lipedema and overweight/obesity experienced significant reductions in body weight, body fat mass, and leg volume. These results suggest that the LCHF diet may have a potential role in reducing adiposity and leg volume, irrespective of baseline body composition. The reduction in body weight was predominantly attributed to a decrease in body fat mass, highlighting the efficacy of the LCHF diet in targeting fat loss, a desirable outcome in the context of obesity treatment. Furthermore, the reduction in visual analog scale (VAS) scores indicates a favorable impact of the LCHF diet on pain perception among the lipedema group. This suggests that the diet intervention not only addresses physiological changes but also contributes to improved subjective well-being in individuals with lipedema.

A meta-analysis conducted by Castellana et al. [30] encompassing adults with overweight and obesity demonstrated the effectiveness of a low-calorie ketogenic diet in reducing body weight, BMI, and waist circumferences. These findings align with the present study, affirming the potential of ketogenic diets in promoting sustainable weight loss. In addition, the comparison between low-carbohydrate ketogenic diet (LCKD) and low-fat diet studies further substantiates the superiority of LCKD in body weight reduction and fat mass loss. The study by Al Aamri et al. [31] showcased the pronounced effects of LCKD in comparison to a low-fat diet. This aligns with our findings and reinforces the potential of LCHF diets to induce significant weight loss. Similarly, a meta-analysis conducted by Zhou et al. [18] and other individual studies [17, 19] supports the notion that LCHF diets are effective in reducing body weight in individuals with overweight and obesity. This consistency in results underscores the robustness of the effect and the potential utility of LCHF diets as a weight management strategy.

Findings from other studies exploring LCHF diets in lipedema patients echo the present study's results. The study involving a 7-week LCHF diet intervention reported reductions in body weight and body circumferences, along with pain reduction. This aligns with our findings, emphasizing the potential of LCHF diets to address both physiological and pain-related aspects of lipedema. In one particular investigation involving nine women diagnosed with lipedema and exhibiting a BMI within the range of 30 to 45 kg/m^2^, a seven-week regimen of LCHF diet was administered. This dietary intervention comprised 70–75% energy from fats, 5–10% energy from carbohydrates, and 20% from protein. The outcome of this dietary intervention manifested as a substantial decrease in body weight by approximately 4.1 kg and a reduction in various body circumferences. However, the change in fat mass was not statistically significant, with a decrease of only 1.5 kg. Additionally, a decrease in pain perception was reported following the LCHF diet. Remarkably, when the participants reverted to their standard diet after the intervention, they observed maintenance of body weight but experienced a recurrence of pain. This led to the conjecture that the pain relief might be attributed to the anti-inflammatory potential of the LCHF diet itself, rather than solely to the weight loss induced by it [21]. The mechanisms underlying pain reduction following the LCHF diet in lipedema patients remain speculative. It is hypothesized that the reduction in leg volume may alleviate hypoxia, leading to a reduction in adipocyte hyperplasia and, subsequently, pain reduction. Further research is needed to elucidate these mechanisms and explore the potential anti-inflammatory effects of LCHF diets in pain management.

Moreover, a case study reported significant body weight reduction and pain improvement following a ketogenic diet in a lipedema woman. In this instance, a ketogenic diet was employed, comprising 1300 kcal daily intake with 30% energy from proteins, 66% energy from fats, and 4% energy from carbohydrates. Furthermore, omega-3 fish oil, along with vitamins C and D, was supplemented in her dietary plan. Remarkably, the woman exhibited a substantial reduction in body weight, amounting to 41 kg, which corresponded to a reduction of approximately 20% in body fat. This dietary intervention also improved pain perception, consequently enhancing her overall quality of life. However, the authors of this case study approached their conclusions with caution, refraining from asserting the ketogenic diet as the exclusive solution for lipedema treatment [22]. Nevertheless, it is notable that our previous research corroborates the pronounced benefits of the ketogenic diet for achieving an effective reduction in body fat, particularly in the lower limbs, in lipedema-affected women [15, 32].

The constraints inherent to our study may arise from the challenge of discerning lipedema from obesity, particularly in cases where patients exhibit subtle lipedema symptoms or a higher degree of obesity. Nevertheless, our study employed rigorous criteria for group selection, and all female participants underwent a thorough evaluation by an angiologist. As a result, the potential for erroneous categorization within the study cohorts has been markedly minimized.

In conclusion, this study contributes valuable insights into the potential benefits of an LCHF diet for reducing weight, body fat mass, and leg volume and managing pain in women with lipedema. The consistent findings across various parameters, coupled with support from existing literature, underscore the potential therapeutic role of LCHF diets in addressing both physiological and subjective aspects of lipedema. Further research is warranted to validate these findings, elucidate underlying mechanisms, and establish the long-term efficacy and safety of LCHF diets in lipedema treatment. Clinical practitioners may consider incorporating LCHF diets as a potential therapeutic strategy for managing lipedema, focusing not only on weight loss but also on reducing pain and improving overall quality of life. The study's findings might encourage more targeted interventions for lipedema, addressing both the physical and psychological aspects of the condition. Researchers and clinicians might collaborate to develop personalized dietary and lifestyle approaches for individuals with lipedema to optimize outcomes in terms of pain relief, body composition, and overall well-being.

## Figures and Tables

**Table 1:** Sample weekly nutritional plan in anti-inflammatory LCHF diet.

Day	Breakfast	Lunch	Dinner
1	Cottage cheese with cucumber, sunflower seeds, and olive oil	Omelette with mushrooms, fresh spinach, tomatoes, and oregano	Curry with chicken and cauliflower and fresh coriander
2	Scrambled eggs with butter, avocado, and radish salad	Lettuce wraps with cream cheese, ham, cherry tomatoes, sprouts, and mixed seeds	Baked feta cheese with zucchini, bell pepper, red onion, olives, herbs, garlic, and olive oil
3	Mozzarella with tomato, fresh basil, and olive oil	Greek yogurt with berry fruits, linen seeds, walnuts, and pumpkin oil	Roasted chicken thighs with cucumber and garlic salad with yoghurt-olive oil sauce
4	Smoked salmon and avocado with tomato-cucumber salad with olive oil	Mixed cheeses (cheddar, Parmesan, and Gouda), walnuts, and black olives salad with olive oil	Fried eggs with green beans in butter and sesame
5	Bell pepper “sandwich” stuffed with mayonnaise, ham, hard cheese, green olives lettuce, and sprouts	Greek salad with feta cheese, green olives, herbs, and olive oil	Stewed turkey with zucchini and tomato in cream onion sauce
6	Boiled eggs with mayonnaise and fresh vegetable salad with olive oil and pumpkin seeds	Grilled halloumi with fresh vegetables (tomato, cucumber, onion, and sprouts) and olive oil	Baked cod with dill and butter and sauerkraut with canola oil
7	Omelette with mascarpone, berry fruits, and coconut flakes	Salad with arugula, cherry tomatoes, olives, parma ham, Parmesan cheese, fresh basil, and olive oil	Grilled chicken and cooked broccoli with herbs dressing with canola oil and seeds

**Table 2:** Comparison of age, anthropometric measurements, body composition, circumferences, and RMR between lipedema (*n* = 28) and patients with overweight/obesity (*n* = 24) at baseline.

Parameter	Lipedema (*n* = 28) mean ± SD/median(Q1, Q3)	Overweight/obesity (*n* = 24) mean ± SD/median (Q1, Q3)	*t*/*Z*	*p* ^*∗*^
Age (years)	39.0 (33.0, 62.0)	49.0 (41.5, 59.0)	1.47	0.14^*∗∗*^
Height (cm)	165.2 ± 7.8	164.1 ± 6.2	−0.55	0.59^*∗*^
Weight (kg)	85.4 ± 16.6	92.1 ± 15.3	1.50	0.14^*∗*^
BMI (kg/m^2^)	33.6 ± 6.2	34.1 ± 4.6	1.71	0.09^*∗*^
LBM (kg)	49.3 (45.5, 55.1)	51.0 (48.1, 57.0)	0.95	0.34^*∗∗*^
PBF (%)	39.9 (35.7, 42.8)	40.5 (36.8, 43.6)	0.53	0.59^*∗∗*^
MBF (kg)	33.6 ± 11.0	37.3 ± 9.1	1.29	0.2^*∗*^
TBW (kg)	38.4 ± 5.5	39.1 ± 5.5	0.45	0.65^*∗*^
VFL	12.6 ± 5.1	11.0 ± 3.5	−1.29	0.2^*∗*^
Waist (cm)	97.8 ± 12.8	106.6 ± 10.6	2.66	0.01^*∗*^
Hips (cm)	115.1 ± 1.0	114.4 ± 8.7	−0.22	0.83^*∗*^
WHR	0.85 ± 0.07	0.93 ± 0.06	4.41	≤0.001^*∗*^
Left thigh (cm)	65.0 ± 7.0	64.8 ± 5.7	−0.12	0.9^*∗*^
Right thigh (cm)	64.9 ± 7.0	64.9 ± 5.9	0.0	1.0^*∗*^
Left calf (cm)	44.3 ± 4.9	42.2 ± 4.0	−1.68	0.1^*∗*^
Right calf (cm)	44.2 ± 5.0	42.4 ± 4.3	−1.35	0.18^*∗*^
Left ankle (cm)	24.5 (23.5, 26.0)	23.0 (22.0, 24.5)	−2.56	0.01^*∗∗*^
Right ankle (cm)	24.0 (23.5, 26.0)	23.3 (22.3, 24.5)	−2.02	0.04^*∗∗*^
Left leg VOL (ml)	11216.5 (10110.0, 14152.0)	10946.0 (8840.0, 12576.0)	−1.79	0.07^*∗∗*^
Right leg VOL (ml)	11910.5 (10208.5, 14259.0)	11239.0 (8991.0, 12185.)	−1.60	0.11^*∗∗*^
RMR (kcal)	1639.0 ± 272.4	1677.3 ± 341.4	0.43	0.67^*∗*^

BMI, body mass index; LBM, lean body mass; PBF, percentage body fat; MBF, mass of body fat; TBW, total body water; VFL, visceral fat level; WHR, waist-hip ratio; VOL, volume; RMR, resting metabolic rate; ^*∗*^*T*-test; ^*∗∗*^Mann–Whitney *U* test; the degree of freedom (df) is 50, except for RMR, where the degree of freedom (df) is 46; *p*  <  0.05 represents statistically significant results.

**Table 3:** Diet composition in lipedema and overweight/obesity groups.

Parameter	Lipedema group (*n* = 28) mean ± SD/me (Q1, Q3)	Overweight/obesity group (*n* = 24) mean ± SD/me (Q1, Q3)	*t*/*Z*	*p* values
Energy value (kcal)	1679.7 ± 129.6	1639.7 ± 132.8	−1.1	0.3^*∗*^
Total protein (g)	88.3 ± 13.6	88.6 ± 7.5	0.1	0.9^*∗*^
Total protein (% kcal)	21.0 ± 2.6	21.7 ± 2.0	1.1	0.3^*∗*^
Total carbohydrates (g)	30.1 (28.1, 33.5)	31.8 (28.3, 33.6)	0.5	0.4^*∗∗*^
Total carbohydrates (% kcal)	6.0 (5.7, 6.8)	6.4 (5.8, 7.0)	0.7	0.7^*∗∗*^
Fiber (g)	8.6 (7.6, 10.8)	8.8 (7.8, 11.9)	0.5	0.5^*∗∗*^
Total fat (g)	133.1 (124.4, 141.1)	131.2 (117.9, 140.0)	−1.0	0.3^*∗∗*^
Total fat (% kcal)	71.7 (70.4, 74.4)	70.7 (68.7, 72.3)	−1.7	0.1^*∗∗*^
SFAs (g)	35.9 ± 8.2	36.2 ± 6.8	0.2	0.9^*∗*^
SFAs (% kcal)	19.1 ± 3.7	19.9 ± 3.7		
MUFA (g)	54.1 ± 10.1	58.4 ± 9.1	1.6	0.1^*∗*^
MUFA (% kcal)	29.1 ± 5.2	32.1 ± 4.3		
PUFA (g)	25.1 ± 5.8	26.2 ± 6.8	0.7	0.5^*∗*^
PUFA (% kcal)	13.5 ± 3.1	14.3 ± 2.9		
n-3 (g)	4.1 ± 1.7	4.5 ± 0.9	1.1	0.3^*∗*^
n-6 (g)	10.6 (9.8, 13.6)	12.4 (10.2, 14.8)	0.9	0.4^*∗∗*^
n-6 to n-3 ratio	3.1 (2.3, 4.1)	2.8 (2.2, 3.4)	−0.9	0.3^*∗∗*^
Cholesterol (mg)	621.3 ± 96.0	581.9 ± 128.9	−1.3	0.2^*∗*^
Sodium (mg)	1860.7 ± 432.4	1947.0 ± 459.5	0.7	0.5^*∗*^
Calcium (mg)	876.1 ± 246.4	925.6 ± 219.9	0.8	0.5^*∗*^
Magnesium (mg)	235.7 (196.6, 274, 1)	241.4 (218.5, 287, 4)	0.9	0.4^*∗∗*^
Potassium (mg)	2254.9 ± 380.9	2347.7 ± 300.1	1.0	0.3^*∗*^
Zinc (mg)	8.8 (7.9, 9.7)	9.0 (7.9, 10.1)	0.2	0.8^*∗∗*^
Phosphorus (mg)	1298.2 ± 272.9	1357.3 ± 149.1	0.9	0.3^*∗*^
Manganese (mg)	1.3 (1.1, 1.7)	1.4 (1.2, 1.7)	0.5	0.6^*∗∗*^
Iron (mg)	9.7 (9.1, 10.4)	9.1 (8.1, 10.3)	−1.4	0.1^*∗∗*^
Copper (mg)	0.9 (0.9,1.1)	1.0 (0.9, 1.2)	0.6	0.6^*∗∗*^
Iodine (*μ*g)	55.4 (43.6, 65.9)	57.4 (42.8, 63.9)	0.0	1.0^*∗∗*^
Retinol (*μ*g)	731.5 ± 207.0	697.0 ± 133.8	−0.7	0.5^*∗*^
Vitamin D (*μ*g)	8.9 ± 3.3	8.5 ± 2.1	−0.4	0.7^*∗*^
Thiamine (mg)	0.8 (0.6, 0.9)	0.8 (0.6, 0.8)	0.5	0.6^*∗∗*^
Niacin (mg)	16.8 (14.6, 23.3)	19.0 (16.8, 23.0)	1.1	0.3^*∗∗*^
Folate (mg)	315.7 ± 59.6	314.8 ± 57.3	−0.1	1.0^*∗*^
Vitamin C (mg)	121.4 (114.2, 137.1)	129.4 (117.7, 144.4)	0.9	0.4^*∗∗*^
Vitamin A (*μ*g)	1186.6 (1071.6, 1349.5)	1132.0 (2921.0, 4205.0)	−0.8	0.4^*∗∗*^
*β*-Carotene (*μ*g)	3333.4 (2613.7, 3852.1)	3414.0 (2921.0, 4205,0)	0.6	0.6^*∗∗*^
Vitamin E (mg)	18.2 (15.2, 20.3)	18.4 (15.1, 21.4)	0.1	0.9^*∗∗*^
Riboflavin (mg)	2.1 (1.9, 2.2)	1.9 (1.6, 2.2)	−1.7	0.1^*∗∗*^
Vitamin B6 (mg)	1.7 (1.5, 1.9)	1.9 (1.6, 2.1)	1.1	0.3^*∗∗*^
Vitamin B12 (mg)	7.5 (6.0, 9.2)	6.9 (5.7, 8.5)	−1.1	0.3^*∗∗*^

SFAs, saturated fatty acids; MUFA, monounsaturated fatty acids; PUFA, polyunsaturated fatty acids; n-6, polyunsaturated fatty acids of the omega-6 family; n-3, polyunsaturated fatty acids of the omega-3 family. ^∗^*T*-test; ^∗∗^Mann–Whitney *U* test; *p*  <  0.05 statistically significant values.

**Table 4:** Comparison of anthropometric and body composition measurements before and after dietary intervention in the study groups.

Parameter	Lipedema group (*n* = 28) mean ± SD/me (Q1, Q3)			Overweight/obesity group (*n* = 24) mean ± SD/me (Q1, Q3)			Differences between baseline and 7 months mean ± SD/me (Q1, Q3)			
	Baseline	7 months	*p*values	Baseline	7 months	*p*values	Lipedema	Overweight/obesity	*t/Z*	*p*values
Weight (kg)	83.8 (76.9, 93.3)	73.4 (64.4, 83.2)	≤0.001^*∗∗*^	91.9 (79.8, 97.8)	77.1 (67.2, 86.5)	<0.001^*∗∗*^	−10.8 (6.4, 14.5)	−11.9 (10.5, 13.8)	1.49	0.14^
BMI (kg/m^2^)	31.4 ± 6.2	27.3 ± 5.1	≤0.001^*∗*^	34.1 ± 4.6	29.3 ± 4.0	≤0.001^*∗*^	−4.1 ± 2.5	−4.8 ± 1.8	1.55	0.12^
LBM (kg)	51.2 ± 6.8	48.7 ± 5.6	<0.001^*∗*^	52.4 ± 7.2	49.7 ± 6.9	<0.001^*∗*^	−2.5 ± 2.5	−2.7 ± 3.0	0.74	0.74^*∗*^
PBF (%)	39.9 (35.7, 42.8)	33.8 (27.8, 37.4)	<0.001^*∗∗*^	40.5 (36.8, 43.6)	33.2 (28.9, 39.3)	<0.001^*∗∗*^	−6.0 (3.4, 8.3)	−6.3 (4.5, 8.4)	0.57	0.57^
MBF (kg)	33.6 (28.4, 39.2)	25.2 (18.8, 30.2)	<0.001^*∗∗*^	36.7 (29.3, 43.1)	25.5 (20.0, 34.5)	<0.001^*∗∗*^	−7.4 (5.5, 12.7)	−9.3 (7.2, 11.9)	1.33	0.18^
TBW (kg)	38.4 ± 5.5	37.6 ± 6.3	0.12^*∗*^	39.1 ± 5.5	37.0 ± 5.3	<0.001^*∗*^	−0.9 ± 2.8	−2.1 ± 2.3	1.72	0.09^*∗*^
VFL	14.0 (8.5, 16.5)	9.5 (4.0, 12.0)	<0.001^*∗∗*^	10.5 (8.0, 13.5)	8.0 (6.0, 10.0)	<0.001∗∗	−3.0 (2.0, 5.0)	−3.0 (2.0, 3.0)	−0.95	0.35^
Waist (cm)	97.8 ± 12.8	86 ± 11.4	≤0.001^*∗*^	106.5 ± 10.6	94.1 ± 11.8	≤0.001^*∗*^	−11.7 ± 6.6	−12.4 ± 6.3	0.38	0.71^*∗*^
Hips (cm)	115.3 (106.5, 122.0)	107.0 (98.0, 111.8)	<0.001^*∗∗*^	114.5 (108.5, 120.0)	104.3 (100.5, 108.3)	<0.001^*∗∗*^	−8.5 (6.3, 11.8)	−8.8 (7.3, 11.5)	0.6	0.55^
WHR	0.8 ± 0.1	0.8 ± 0.1	<0.001^*∗*^	0.9 ± 0.1	0.9 ± 0.1	0.02^*∗*^	0.0 ± 0.0	0.0 ± 0.1	−0.43	0.67^*∗*^
Left thigh (cm)	65.0 ± 7.0	59.2 ± 5.6	≤0.001^*∗*^	64.8 ± 5.7	59.0 ± 5.3	≤0.001^*∗*^	−5.9 ± 3.1	−5.9 ± 2.2	−0.03	0.98^*∗*^
Right thigh (cm)	64.9 ± 6.9	58.9 ± 5.6	≤0.001^*∗*^	64.9 ± 5.9	59.0 ± 5.5	≤0.001^*∗*^	−5.9 ± 3.3	−5.9 ± 2.4	−0.09	0.93^*∗*^
Left calf (cm)	44.0 (40.3, 46.5)	40.3 (37.5, 43.0)	<0.001^*∗∗*^	42.0 (40.3, 44.0)	39.3 (37.5, 41.3)	<0.001^*∗∗*^	−3.5 (2.8, 4.8)	−2.8 (1.8, 4.0)	−1.8	0.07^
Right calf (cm)	44.3 (40.3, 47.0)	41.3 (37.8, 43.0)	<0.001^*∗∗*^	42.3 (39.3, 44.8)	39.5 (37.5, 41.0)	<0.001^*∗∗*^	−3.0 (2.0, 4.5)	−2.5 (1.8, 3.3)	−1.56	0.12^
Left ankle (cm)	24.5 (23.5, 26.0)	23.8 (22.5, 25.0)	<0.001^*∗∗*^	23.0 (22.0, 24.5)	23.0 (22.3, 24.0)	0.09^*∗∗*^	−1.0 (0.5, 1.5)	−0.5 (0.0, 0.5)	−3.2	<0.01^
Right ankle (cm)	24.0 (23.5, 26.0)	23.3 (22.5, 24.8)	<0.001^*∗∗*^	23.3 (22.3, 24.5)	23.0 (22.2, 24.0)	0.04^*∗∗*^	−1.0 (0.5, 1.8)	−0.5 (0.0, 0.8)	−2.72	<0.01^
Left leg VOL (ml)	11216.5 (10110.0, 14152.0)	9707.5 (8785.0, 11849.8)	<0.001^*∗∗*^	10946.0 (8840.0, 12576.0)	8725.5 (7421.0, 10524.0)	<0.001^*∗∗*^	−1395.5 (811.5, 2614.0)	−1443.2 (1066.0, 2057.5)	−0.09	0.93^
Right leg VOL (ml)	11910.5 (10208.5, 14259.0)	10063.0 (8543.5, 12556.5)	<0.001^*∗∗*^	11239.0 (8991.0, 12185.0)	9503.8 (7578.0, 10892.0)	<0.001^*∗∗*^	−1524.0 (865.0, 2357.5)	−1487.5 (1031.5, 1961.5)	−0.07	0.94^
VAS	4.6 ± 2.6	3.0 ± 2.3	<0.001^*∗*^	—	—	—	—	—	—	—

BMI, body mass index; LBM, lean body mass; PBF, percentage body fat; MBF, mass of body fat; TBW, total body water; VFL, visceral fat level; WHR, waist-hip ratio; VOL, volume; RMR, resting metabolic rate; ^*∗*^*T*-test; ^*∗∗*^Wilcoxon test; ^Mann–Whitney *U* test; df = 23 (overweight/obesity); df = 27 (lipedema); *p*  <  0.05 statistically significant values.

## Data Availability

All data used in this study are available upon request from the corresponding author.

## Supplementary Materials

Supplementary Materials Table S1: comparison of vitamin and mineral intake with Polish recommended levels (Supplementary Materials).
