# Supplementary material for: The Benefits of Low-Carbohydrate, High-Fat (LCHF) Diet on Body Composition, Leg Volume, and Pain in Women with Lipedema
Source: J Obes. 2023 Nov 18;2023:5826630. doi: 10.1155/2023/5826630 (PMC10676278; doi:10.1155/2023/5826630)
Supplement: Supplementary Materials — Table S1: comparison of vitamin and mineral intake with Polish recommended levels (Supplementary Materials). [file 5826630.f1.docx]

**Table S1** Comparison of vitamin and mineral intake with Polish recommended levels

| **Nutrient** | **Recommended level/day** | **Lipedema** | | **Overweight/obesity** | |
| --- | --- | --- | --- | --- | --- |
|  |  | **Intake from the study**  **[median]** | **Compliance with recommendations [%]** | **Intake from the study**  **[median]** | **Compliance with recommendations [%]** |
| Iodine [μg/day] | 150 | 55,4 | *36,9* | 57,4 | *38,3* |
| Iron [mg/day] | 18 (≤50 y.)  10 (>50 y.) | 9,6  9,9 | *53,3*  *99,0*  *(mean = 76,2)* | 9,4  8,5 | *52,2*  *85,0*  *(mean = 68,6)* |
| Vit. D [μg/day] | 15 | 8,9 | *59,1* | 8,5 | *56,9* |
| Potassium [mg/day] | 3500 | 2254,9 | *64,4* | 2347,7 | *67,1* |
| Thiamine [mg/day] | 1,1 | 0,8 | *68,2* | 0,8 | *68,4* |
| Manganese [mg/day] | 1,8 | 1,3 | *73,6* | 241,4 | *75,4* |
| Magnesium [mg/day] | 320 | 235,7 | *73,7* | 1,4 | *77,8* |
| Folate [μg/day] | 400 | 315,7 | *78,9* | 314,8 | *78,7* |
| Calcium [mg/day] | 1000 | 876,1 | *87,6* | 925,6 | *92,6* |
| Copper [mg/day] | 0,9 | 0,9 | *105,0* | 1,0 | *110,6* |
| Zinc [mg/day] | 8 | 8,8 | *110,2* | 9,0 | *112,3* |
| Niacin [mg/day] | 14 | 16,8 | *120,3* | 1947,0 | *129,8* |
| Sodium [mg/day] | 1500 | 1860,7 | *124,0* | 19,0 | *135,6* |
| Vit.B6 [mg/day] | 1,3 | 1,7 | *131,8* | 1,9 | *143,8* |
| Vit. C [mg/day] | 75 | 121,4 | *161,9* | 1132,0 | *161,7* |
| Vit. A [μg/day] | 700 | 1186,6 | *169,5* | 1,9 | *171,8* |
| Phosphorus [mg/day] | 700 | 1298,2 | *185,5* | 129,4 | *172,5* |
| Riboflavin [mg/day] | 1,1 | 2,1 | *188,6* | 1357,3 | *193,9* |
| Vit. E [mg/day] | 8 | 18,2 | *227,6* | 18,4 | *229,5* |
| Vit.B12 [μg/day] | 2,4 | 7,5 | *312,0* | 6,9 | *288,3* |
